# AGE-RELATED MICROAGGRESSIONS: A FOLLOW-UP DESCRIPTIVE STUDY

**DOI:** 10.1093/geroni/igad104.2127

**Published:** 2023-12-21

**Authors:** Hannah Lewis, Stephanie Patt, Luke Gietzen, Jeffrey Buchanan

**Affiliations:** University of Minnesota, Mankato, Minnesota, United States; Minnesota State University, Mankato, Mankato, Minnesota, United States; University of North Dakota, Fargo, North Dakota, United States; Minnesota State University, Mankato, Mankato, Minnesota, United States

## Abstract

Age-related microaggressions are forms of ageist discrimination that occur during day-to-day interactions. The aim of this study was to identify common types of age-related microaggressions as well as to determine how negative affect influences emotional reactions to microaggressions. Using an online survey, participants (n = 200) were asked if they had experienced any of the 20 most common examples of age-related microaggressions reported in previous research (Gietzen et al, 2022). Follow up questions inquired about the frequency, emotional reactions, and behavioral responses to these microaggressions. Participants also rated their physical health and completed the Positive and Negative Affect Scale (PANAS; Watson et al., 1988). The results indicated that participants were familiar with these microaggressions 53% of the time. Participants also reported having negative reactions to 43% of these microaggressions. The frequency of negative emotional responses to microaggressions was significantly correlated with scores on the negative affect subscale of the PANAS (r = .34, p < .001) and with ratings of perceived physical health (r = -.32, p = .002). Finally, an analysis of the 20 survey items revealed that two items were “highly impactful” microaggressions, defined as microaggressions that older adults reported experiencing often and reported having a negative emotional reaction to at least one-third of the time. The results of the study provide further insight into what age-related microaggressions look like, and how older adults experience these interactions.

